# RNA sequencing for ligature induced periodontitis in mice revealed important role of S100A8 and S100A9 for periodontal destruction

**DOI:** 10.1038/s41598-019-50959-7

**Published:** 2019-10-11

**Authors:** Shogo Maekawa, Satoru Onizuka, Sayaka Katagiri, Masahiro Hatasa, Yujin Ohsugi, Naoki Sasaki, Kazuki Watanabe, Anri Ohtsu, Rina Komazaki, Kohei Ogura, Tohru Miyoshi-Akiyama, Takanori Iwata, Hiroshi Nitta, Yuichi Izumi

**Affiliations:** 1 Department of Periodontology, Graduate School of Medical and Dental Sciences, Tokyo Medical and Dental University, Tokyo, Japan; 2 Institute of Advanced Biomedical Engineering and Science, Tokyo Women’s Medical University, Tokyo, Japan; 3 Division of Periodontology, Kyushu Dental University, Fukuoka, Japan; 4 Pathogenic Microbe Laboratory, Research Institute, National Center for Global Health and Medicine, Tokyo, Japan; 5 Advanced Health Care Science Research Unit, Institute for Frontier Science Initiative, Kanazawa University, Ishikawa, Japan; 6 Behavioral Dentistry, Graduate School of Medical and Dental Sciences, Tokyo Medical and Dental University, Tokyo, Japan; 7 Oral Care Perio Center, Southern TOHOKU Research Institute for Neuroscience, Southern TOHOKU General Hospital, Fukushima, Japan

**Keywords:** Sequencing, Bacterial infection

## Abstract

Periodontitis is an inflammatory disease caused by pathogenic oral microorganisms that induce the destruction of periodontal tissue. We sought to identify the relevant differentially expressed genes (DEGs) and clarify the mechanism underlying the rapid alveolar bone loss by using ligature-induced periodontitis in mice. A silk ligature was tied around the maxillary left second molar in 9-week-old C57BL/6 J male mice. *In-vivo* micro-CT analysis revealed that ligation induced severe bone loss. RNA-sequencing analysis, to examine host responses at 3 days post-ligation, detected 12,853 genes with fragments per kilobase of exon per million mapped reads ≥ 1, and 78 DEGs. Gene ontology term enrichment analysis revealed the expression profiles related to neutrophil chemotaxis and inflammatory responses were significantly enriched in the ligated gingiva. The expression levels of innate immune response-related genes, including *S100a8* and *S100a9*, were significantly higher in the ligated side. S100A8 was strongly detected by immunohistochemistry at the attached epithelium in ligated sites. Inhibition of *S100A8* and *S100A9* expression revealed that they regulated *IL1B* and *CTSK* expression in Ca9-22 cells. Thus, innate immune response-related molecules might be associated with the burst-destruction of periodontal tissue in ligature-induced periodontitis. Especially, S100A8 and S100A9 may play an important role in alveolar bone resorption.

## Introduction

Periodontal disease is an inflammatory disease caused by microorganisms that colonize around the teeth^1,2^. Inflamed and damaged tissue, including the gingival epithelium and periodontal connective tissue, produce various inflammatory cytokines^3,4^. These inflammatory cytokines break down the homeostasis of periodontal tissues, activate fibroblasts and osteoclasts, and finally induce loss of connective tissue attachment and alveolar bone^5,6^.

A lot of studies support the concept of a synergistic microbial community acting as the modulator of systemic health. A disruption in the balance of symbiotic microbiota in the small intestine can lead to several diseases including diabetes, rheumatoid arthritis, and inflammatory bowel disease^7^. Based on this theory, Hajishengallis *et al*. proposed a new pathogenesis model of periodontal disease^8^ and established a novel ligature-induced periodontitis model in mice^9^. This model enables the local longitudinal accumulation of anaerobic bacteria and represents rapid bone loss within several days compared to previous models that required oral gavage with high doses of specific bacteria^10,11^ and injection of lipopolysaccharide (LPS)^12^.

In this decade, next-generation sequencing methods for analyzing RNA sequences have been developed and widely performed. RNA sequencing (RNA-seq) is superior in its ability to identify differentially expressed genes (DEGs), alternative gene spliced transcripts, post-transcriptional modifications, gene fusions, and mutations in gene expression^13,14^.

Many studies in humans have tried to elucidate the mechanisms underlying the rapid breakdown of periodontal tissue; however, there has been very little success owing to the complexity in pathogenesis of periodontal disease, variances of patients’ genome, and varieties of environmental factors. Although distinct from human beings, mice are widely used as models for assessing disease pathogenesis owing to their homologous genome in same species. This study aimed to identify the DEGs, and investigated the mechanisms underlying rapid periodontal tissue destruction using RNA-seq analysis of ligature-induced periodontitis in mice.

## Results

### Ligation-induced alveolar bone loss around the second molar tooth

**Figure 1:**
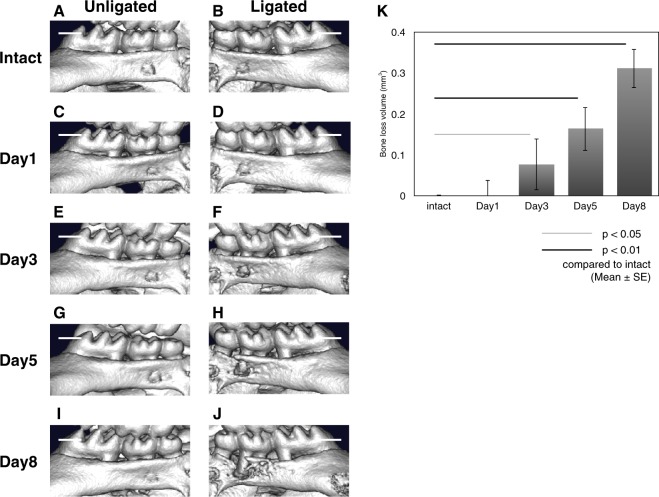
Ligation-induced alveolar bone loss around the second molar. The 3D images of unligated side (intact (**A**), day 1 (**C)**, day 3 (**E**), day 5 (**G**), day 8 (**I**)) and ligated side (intact (**B**), day 1 (**D**), day 3 (**F**), day 5 (**H**), day 8 (**J**)) are shown. The graph shows bone loss volume (mm^3^) at the indicated time points. Bone loss was seen to initiate from the distal root and become wide-spread at 3 days post-ligation. Bone loss volume significantly increased from 3 days post-ligation (**K**). Scale bar = 0.5 mm. Data are represented as the mean ± SE. Thin line: p < 0.05, thick line: *p* < 0.01 compared to intact.

Micro CT imaging showed that neither unligated nor ligated 2^nd^ molar displayed bone loss before and 1 day after ligation (Fig. 1A–D). Alveolar bone loss was initiated around 3 days post-ligation (Fig. 1E,F). Bone loss started from the distal root and gradually spread therefrom (Fig. 1E–J). At 8 days post-ligation, severe intrabony defect was observed around the ligated 2^nd^ molar (Fig. 1I,J). Bone loss volume was gradually and significantly increased from 3 days after ligation (Fig. 1K).

### Hematoxylin and eosin (HE) staining and Tartrate-resistant acid phosphatase (TRAP) and alkaline phosphatase (ALP) staining at 8 days after ligation

**Figure 2:**
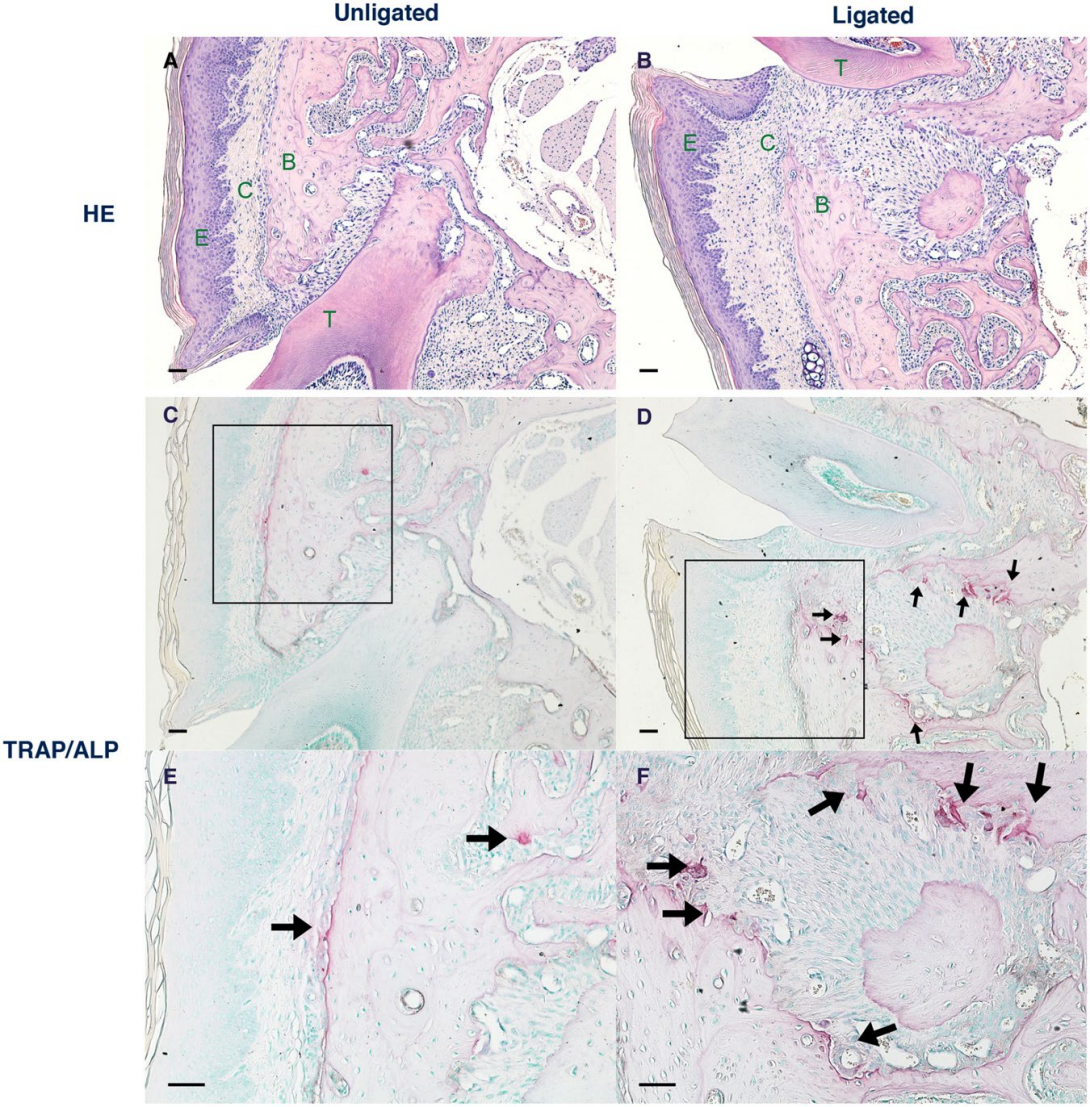
Histological analysis at 8 days after ligation. Hematoxylin and eosin staining (unligated (**A**) and ligated (**B**) side) and TRAP/ALP staining (unligated (**C**) and ligated (**D**) side) are shown. Enlarged views, of the boxed area of unligated (**C**) and ligated (**D**) side, are shown in (**E**,**F**), respectively. TRAP-positive multinucleate cells were induced around the periodontal ligament in ligated side. The arrows in sections indicate osteoclasts stained with TRAP, adjacent to the alveolar bone. Black scale bar = 50 μm. HE, hematoxylin and eosin; TRAP, tartrate-resistant acid phosphatase; ALP, alkaline phosphatase; E, epithelial tissue; C, connective tissue; B, alveolar bone; T, tooth.

HE staining at 8 days post ligation also verified alveolar bone loss around the root in the ligated side, but not in the unligated side (Fig. 2A,B). Further, TRAP-positive multinucleate cells were induced around periodontal ligament in ligated side (Fig. 2C–F) at 8 days post-ligation.

### Gene profile in gingival tissue at 3 days post-ligation analyzed by RNA-seq

**Figure 3:**
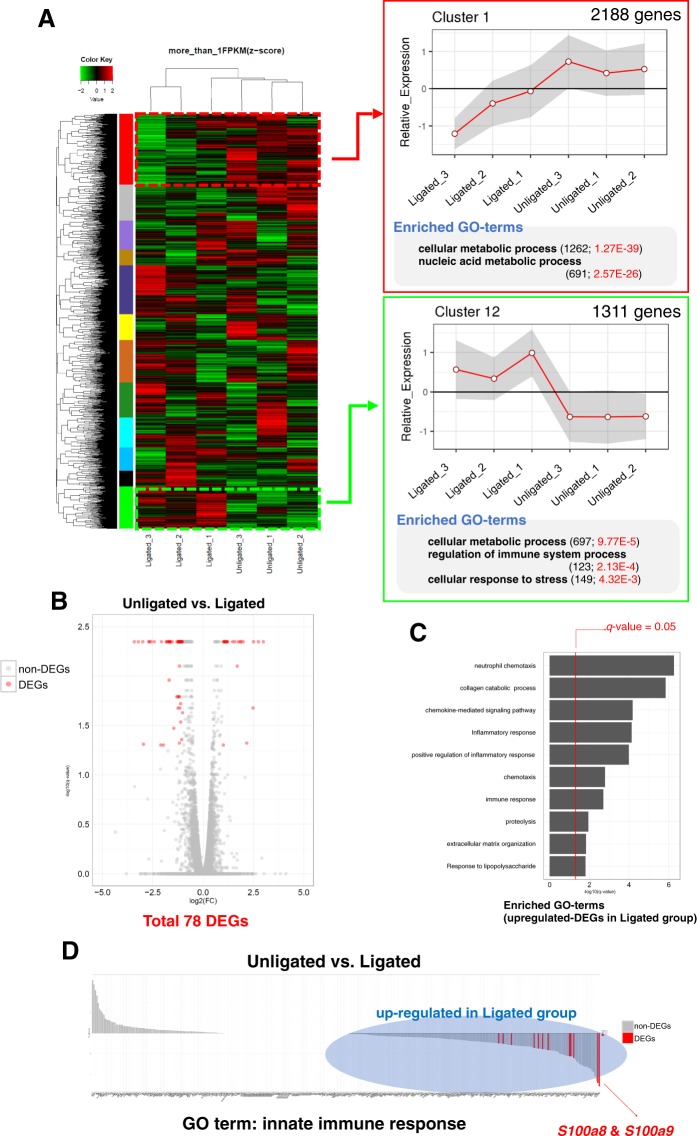
Gene profile in gingival tissue analyzed by RNA sequencing. RNA-seq detected 12,853 genes (FPKM ≥ 1.0), which were categorized into 12 patterns by hierarchical clustering approach (**A**; left). Cluster 1, which consists of genes down-regulated in the ligated gingiva, and Cluster 12, which consists of genes up-regulated in the ligated gingiva, were analyzed using DAVID to determine specific enriched GO terms. The figure shows the representative significantly enriched GO terms (Benjamini-Hochberg <0.05) (**A**; right). Statistical analysis revealed 78 (55 up-regulated and 23 down-regulated) DEGs between the ligated and unligated gingiva, using the criteria of *q*-value < 0.05 and fold change ≥2.0. The red and gray plots represent DEGs and non-DEGs, respectively (**B**). Top 10 enriched GO terms in the up-regulated DEGs in ligated gingiva were determined by DAVID. Right side of the red line designates Benjamini-Hochberg <0.05 (**C**). The bar plot shows fold change (on a log 2 scale) between the ligated and unligated gingiva for innate immune response-related genes. The red and gray bars represent DEGs and non-DEGs, respectively (**D**).

Illumina MiSeq system generated 7,674,282–12,712,894 (Ave. 9,377,592) pair-ended 101-bp reads per sample (n = 3). The base quality of sequencing reads was confirmed and analyzed using the quality control tool FastQC (Supplementary Fig. S1). TopHat2-Cufflinks pipeline was employed to compare the uniquely mapped reads with mouse reference genome (mm10) and to quantify RefSeq gene expression as the unit of fragments per kilobase of exon per million mapped reads (FPKM). Almost all sequencing reads (89.8–90.8%) were uniquely mapped to mm10 (Supplementary Fig. S2A). Distribution of FPKM for all genes (24,247 genes) was consistent across all samples (Supplementary Fig. S2B). Although these results suggested our sequencing data to be high quality, majority of the genes showed low expression (FPKM < 1.0). To remove the low-expression genes between the two groups, only RefSeq genes, expressed in at least one of the groups with FPKM ≥ 1, were used for this study, which consisted of 12,853 genes. Among these genes, 12 different gene clusters were detected exhibiting distinct expression patterns (Fig. 3A). The genes in clusters 1 (showing decreased relative expression levels in the ligated gingiva compared to that in unligated gingiva) and 12 (showing increased relative expression levels in the ligated gingiva compared to that in unligated gingiva) were significantly enriched by specific gene ontology (GO) terms. GO terms associated with metabolic process were detected in both gene clusters. Genes in cluster 12 possessed specific GO-terms related to the regulation of immune system processes and cellular response to stress (Fig. 3A).

To detect the potential key regulators during alveolar bone loss by ligation, we analyzed the DEGs (fold change ≥2, *q*-value (the false discovery rate (FDR) with Benjamini-Hochberg correction) < 0.05) between ligated and unligated gingiva, and identified 78 genes (Fig. 3B and Supplementary Table S1). To investigate the global function of genes up-regulated by ligation, GO enrichment analysis was performed, and top 10 significantly enriched GO biological process terms (*q*-value < 0.05) were identified for 55 up-regulated DEGs. Inflammation-related GO terms, such as “*neutrophil chemotaxis*”, “*inflammatory response*”, “*positive regulation of inflammatory response*”, and “*immune response*” were detected for the genes significantly up-regulated in ligated gingiva (Fig. 3C). Moreover, since these results were based on samples at just 3 days post-ligation, the immune responses must be innate immune responses. Thus, we analyzed the innate immune response-related genes. Figure 3D shows that majority of the genes categorized under the GO term of innate immune response which were up-regulated in ligated gingiva. Among the innate immune response-related genes, *S100a8* and *S100a9* were dramatically increased in ligated gingiva compared to that in unligated gingiva. Therefore, we focused on the innate immune response, especially *S100a8* and *S100a9*, as potential key genes affecting the rapid and severe alveolar bone loss caused by ligation.

### mRNA expression levels by qPCR in DEGs

**Figure 4:**
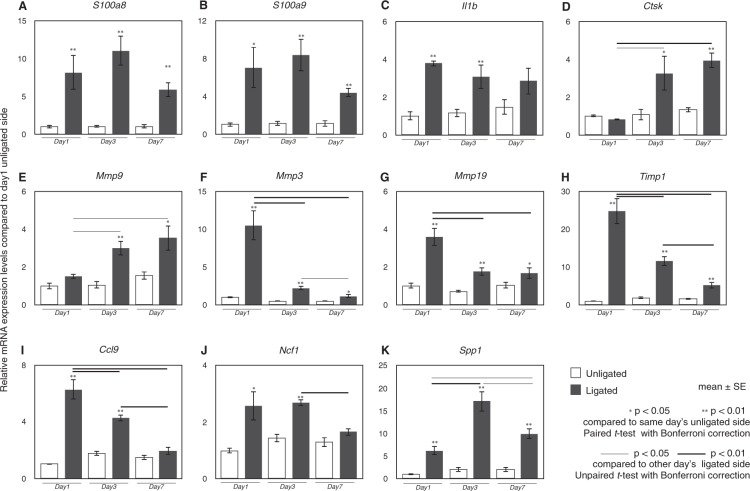
The mRNA expression levels of DEGs at 1, 3, and 7 days after ligation. The mRNA expression levels of DEGs were validated by qPCR after 1, 3, and 7 days of ligation. Relative expression levels of *S100a8* (**A**), *S100a9* (**B**), *Il1b* (**C**), *Ctsk* (**D**), *Mmp9* (**E**), *Mmp3* (**F**), *Mmp19* (**G**), *Timp1* (**H**), *Ccl9* (**I**), *Ncf1* (**J**), and *Spp1* (**K**) are shown. The mean mRNA expression levels in the unligated gingiva at day 1 were set as “1”. Data are shown as the mean ± SE. *p < 0.05, **p < 0.01 compared to same-day unligated side. Thin line: *p* < 0.05, thick line: *p* < 0.01 compared to other day’s ligated side.

Following the results of RNA-seq analysis, mRNA expression was validated by quantitative PCR (qPCR). Among 78 DEGs, we focused on the genes related to innate immune response, damage-associated molecular patterns (DAMPs), and periodontal tissue metabolism. *S100a8* and *S100a9* were highly expressed at all time-points in the ligated gingiva (Fig. 4A,B). *Il1b* expression level was higher at all experimental days in the ligated side compared to that in the unligated side (Fig. 4C). *Ctsk* and *Mmp9* expression consistently increased from 3 day post-ligation (Fig. 4D,E), while *Mmp3*, *Mmp19*, *Timp1*, and *Ccl9* expression gradually decreased after an increase at 1 day post-ligation (Fig. 4F–I). While *Ncf1*, a marker of neutrophil, was increased at 1 and 3 days post-ligation, it was decreased at 7 days post-ligation (Fig. 4J). *Spp1* expression level was higher at all days in the ligated side compared to that in the unligated side (Fig. 4K).

### Detection of S100A8 by immunohistochemistry at 3 and 8 days post-ligation

**Figure 5:**
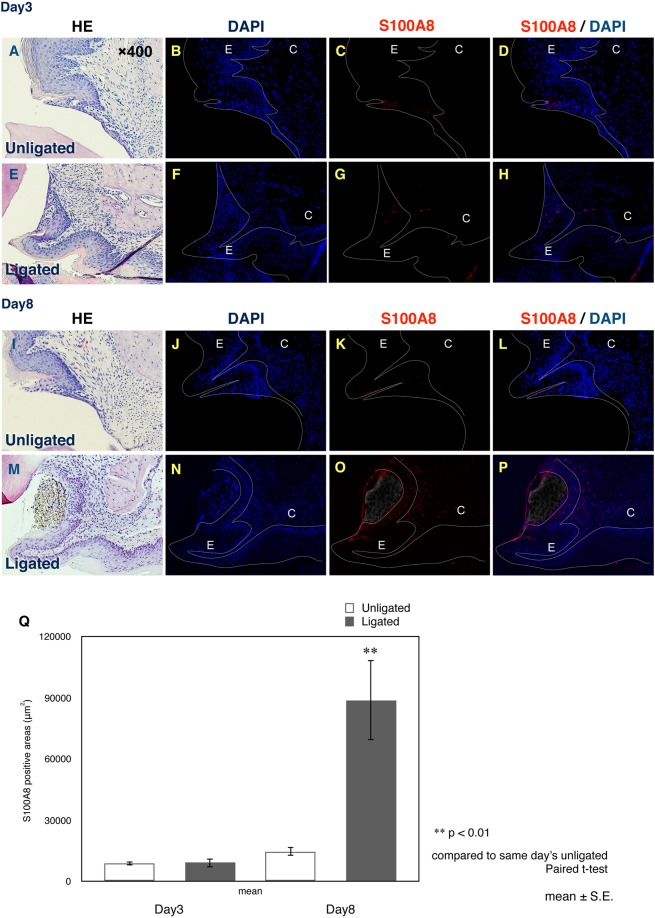
Histological analysis of periodontal tissue at 3 days and 8 days post-ligation. Hematoxylin-eosin staining (3-day unligated (**A**), 3-day ligated (**E**), 8-day unligated (**I**), 8-day ligated (**M**)), nuclear staining with DAPI (3-day unligated (**B**), 3-day ligated (**F**), 8-day unligated (**J**), 8-day ligated (**N**)), and immunohistochemistry of S100A8 (3-day unligated (**C**), 3-day ligated (**G**), 8-day unligated (**K**), 8-day ligated (**O**)) of periodontal tissue are shown. Merged images with S100A8 and DAPI (3-day unligated (**D**), 3-day ligated (**H**), 8-day unligated (**L**), and 8-day ligated (**P**)) are also shown. S100A8 expression areas were evaluated and S100A8-positive area in ligated side at 8 days post-ligation was found to be significantly increased than that of unligated side (**Q**). Data are shown as the mean ± SE. **p < 0.01 compared to same-day unligated side; E, epithelial tissue; C, connective tissue. Epithelium is outlined by white line.

Through immunohistochemistry, S100A8 expression at 3 days post-ligation could be identified in the attached epithelium of the ligated side, as well as of the unligated side (Fig. 5A–H). At 8 days post-ligation, S100A8 could be identified obviously in the connective tissue present only in the ligated gingiva (Fig. 5I–P). Moreover, at this time point, the expression of S100A8 was significantly increased in the ligated gingiva compared to that in the unligated gingiva (Fig. 5Q).

### *S100A8* and *S100A9* knockdown in Ca9-22 cells

**Figure 6:**
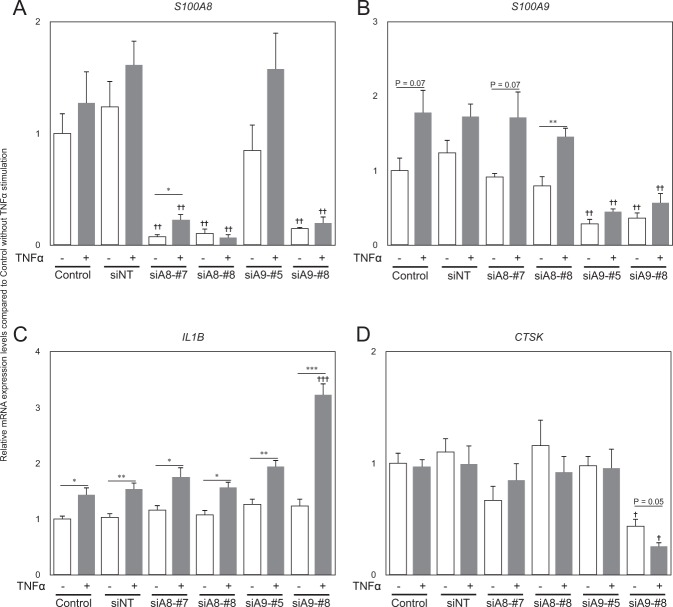
S100A8 and S100A9 knockdown in Ca9-22 cells. *S100A8* (**A**), *S100A9* (**B**), *IL1B*(**C**), and *CTSK* (**D**) expression in Ca9-22 cells. Control cells were cultured in medium with transfection reagent. siNT, siRNA for non-target negative control; siA8-#7 and siA8-#8, siRNA for S100A8; siA9-#5 and siA9-#8, siRNA for S100A9. *p < 0.05, **p < 0.01, ***p < 0.001. ^†^*p* < 0.05, ^††^*p* < 0.01, ^†††^*p* < 0.001 compared to control samples.

Each of the two siRNAs targeting S100A8 (siA8-#7 and siA8-#8) suppressed *S100A8* expression by almost 90%. While one of siRNA targeting S100A9 (siA9-#5) suppressed *S100A9* expression specifically, the other one (siA9-#8) did both *S100A8* and *S100A9* expression probably because the siRNA can target both S100A8 and S100A9 mRNAs (Fig. 6A,B). Stimulation of Ca9-22 cells with TNF-α significantly increased *IL1B* expression, whereas *CTSK* expression remained unchanged (Fig. 6C,D). Surprisingly, Ca9-22 cells with *S100A8*/*S100A9* double-knockdown showed dramatic changes in expression of *IL1B* and *CTSK* genes. *IL1B* gene expression, after 24 h of TNF-α stimulation increased almost 3 times compared to that in control cells. On the other hand, *CTSK* expression was significantly down-regulated by the double-knockdown.

## Discussion

There are several hypotheses regarding the destruction process and its rate in periodontal tissues. Socransky *et al*. proposed the random disease model^15^, based on the concept that periodontal disease progression consisted of burst-destruction followed by a remission of the disease, as opposed to continuous destruction. In our model, bone resorption was initiated at 3 days post-ligation, and TRAP-positive cells, which mark osteoclasts, were increased around the alveolar bone at 8 days post-ligation. These findings were consistent with a previous report about ligature-induced periodontitis in mice^9^. Thus, ligation seems to trigger a burst of destruction of the periodontal tissue, and this model may be similar to periodontitis in activated progression phase. Sawle *et al*. in 2016 had shown that human gingival tissue, from patient with periodontitis, has specific gene markers such as *HCLS1*, *IKZF3*, *ETS1*, *HGLH2*, *POU2F*, and *VAVI*^16^. They had also shown limited molecular dissimilarities in gingival tissue between chronic and aggressive periodontitis^17^. However, we identified 55 up-regulated DEGs related to immune responses and neutrophil chemotaxis in the ligated gingiva. The differences in detected genes between our model and human studies might be attributed to the difference in sampling time point. Our model is based on innate immune response, whereas human periodontitis is based on acquired immune response. In addition, in human samples, it is difficult to identify whether the samples were from the site of active or inactive disease.

Generally, inflammation is initiated by pathogen-associated molecular patterns (PAMPs) such as fimbriae, various kinds of enzymes, and LPS from microorganisms. In the several past years, multiple diseases have been associated with the immune system owing to dysbiosis^18^. Hajishengallis *et al*. had proposed dysbiosis also plays a role in periodontal disease^19^. Once plaque accumulation and calculus deposition occur on the tooth surface, local dysbiosis is initiated around the tooth, which in turn induces gingival inflammation, leading to periodontitis. An endogenous inhibitor of neutrophil adhesion inhibits IL-17-mediated inflammatory bone loss^20^. Moreover, Th17 cells, converted from Foxp3^+^ T cells, play important roles for bone resorption and host immune response against microbial infection^21^. Therefore, periodontal disease progression is modulated in a complex manner by both innate and acquired immune response.

DAMPs, also known as alarmins, are molecules released by damaged cells under conditions of necrosis. These act as endogenous danger signals to promote and exacerbate the inflammatory response^22^. The best-known DAMPs are high mobility group box-1 (HMGB1), S100A8 (MRP8, calgranulin A), S100A9 (MRP14, calgranulin B), and serum amyloid A. S100A8 and S100A9 are calcium-binding proteins expressed in cells of myeloid origin, and are released in case of cell damage or activation of phagocytes and monocytes. S100A8 and S100A9 interact with the receptor for advanced glycation end products and Toll-like receptors, and activate immune cells and vascular endothelium^23^. Currently, S100-family proteins are being considered as new targets for the treatment of osteoarthritis and cancer^24,25^. Interestingly, Nishii *et al*. reported the distribution and expression of S100A8 and S100A9 in gingival epithelium. While S100A8 and S100A9 expression was up-regulated in junctional epithelium in presence of oral microflora, only S100A8 was expressed there in absence of microorganisms^26^. Our results also showed that S100A8 expression was limited to the attached epithelium without ligation, which is consistent with the findings in a previous report^26^. Furthermore, our histological results showed S100A8 expression level to be significantly up-regulated in ligated gingiva at 8 days post-ligation, and it was not only within the attached epithelium but also in the connective tissue. These findings were supported by our qPCR results. Both *S100a8* and *S100a9* expression in ligated gingiva were dramatically increased at days 1, 3, and 7. S100A8 and S100A9 are known as the major proteins in neutrophils and monocytes^27^. Ncf1, also known as neutrophil cytosolic factor 1, is a marker of neutrophil and a key factor in the production of reactive oxygen species^28^. mRNA expression of *Ncf1* was only up-regulated at days 1 and 3 in ligated gingiva and down-regulated at day 7. These differences of mRNA expression pattern among *S100a8*, *S100a9*, and *Ncf1* suggested that the significant high expression level of *S100a8* and *S100a9* were not only from neutrophils but also from inflamed epithelial tissues. Moreover, our inhibition study, using Ca9-22 cells and siRNA, revealed *S100A8* and *S100A9* in epithelial cells to regulate *IL1B* and *CTSK* expression. Cathepsin K is a member of the papain family of cysteine proteases and is highly expressed by mature osteoclasts. Cathepsin K degrades type I collagen, which is the major component of bone matrix, and is considered to be a new treatment target for osteoporosis^29^. There have only been a few reports about Cathepsin K expression in epithelial tissue; therefore, our siRNA experiment highlighted the interaction between S100A8/S100A9 and Cathepsin K in epithelium. Moreover, our results suggest this interaction to possibly play an important role in the progression of periodontal disease due to collagen degradation of Cathepsin K. Calprotectin, a heterodimer of S100A8 and S100A9, has been reported to be released at the inflammation site and induce IL-6 production in human gingival fibroblasts to promote the progression of periodontal disease^30^. Our immunohistochemistry results showed high expression of S100A8 in both epithelial and connective tissues in the ligated gingiva. Taken together, S100A8 and S100A9, in periodontal tissue, have essential roles for the progression of periodontitis.

In addition, our results indicated the breakdown of connective tissue homeostasis. After ligation, expression of *Il1b*, the gene encoding IL-1β is crucial for immune responses against injuries and microbial infections^31^, was elevated and innate immune response initiated, especially for collagen remodeling functional genes^32^. Our results showed *S100A8* and *S100A9* suppressed *IL1B* in gingival epithelial cells; however, *Il1b* expression level in periodontal tissue, including epithelial and connective tissues, was elevated in mouse ligature model, thereby implying that non-epithelial tissues may play a strong role in the induction of inflammatory molecules such as IL1B. In addition, *Mmp3*, *Mmp19* and *Timp1* were elevated immediately after ligation and got decreased day by day, however, *Mmp9* expression in ligated gingiva increased at days 3 and 7 after ligation. Mmp9 plays an important role in the degradation of gelatin, collagen, and extracellular matrix and is a key modulator of angiogenesis^33^. Therefore, Mmp9 elevation in gingival tissue also participates in inflammation in mouse model of ligature-induced periodontitis. Hiyari *et al*. reported that cxcl family members might partially mediate LPS-induced periodontitis in mice^34^. *Ccl9*, which is expressed in macrophages constitutively^35^ and is known as an activator for osteoclasts^36^, is increased at days 1 and 3. *Spp1*, encoding gene for Osteopontin, also known as a possible factor for activated osteoclasts binding to bone^37^, was increased and peaked at day 3. Taken together, up-regulation of these genes support the breakdown of homeostasis in periodontal tissues, and the rapid bone loss seemed to be associated with DAMPs and innate immune response.

A tooth consists of enamel, dentin, pulp, and cementum. Under healthy conditions, only enamel is exposed to the oral cavity. Unlike skin and mucosa, enamel never shows turn over; thus, in absence of tooth brushing, a biofilm is easily developed around the teeth. Additionally, the junction between tooth enamel and attached epithelium is the most important factor in the formation of an epithelial barrier. In periodontal disease, tooth root becomes exposed to the oral cavity due to loss of attachment, and long epithelial attachment is formed. Therefore, protection of the attached epithelium is thought to be key to prevent periodontal disease. The findings that S100A8 was expressed only in the attached epithelium without any stimulation, and up-regulated in periodontal tissues of mice exhibiting rapid bone loss, suggest S100A8 as a possible key target for periodontal treatment.

A tooth plays an important role in occlusion and occlusal force. When excessive occlusal force is loaded against the teeth, occlusal trauma affects the periodontium, especially cementum, periodontal ligament, and alveolar bone^38–45^. Rapid alveolar bone loss is generally associated with cases of combined inflammation and occlusal trauma. While the effects of mechanical forces against fibroblasts such as periodontal ligament cells^46^ have been reported, there still remain unknown details about the relationships between DAMPs and occlusal forces against periodontal tissue.

Aggressive periodontitis is characterized by rapid attachment loss and alveolar bone loss^47^. Patients suffering from aggressive periodontitis have severe alveolar bone loss and periodontal inflammation, even at a young age, and with less amount of dental plaque. Several studies examining aggressive periodontitis have focused on host responses determined by the genetic background, such as SNPs^48^, GWAS results^49^, and NOD2 mutations^50^. However, the detailed mechanism still remains unclear. A new classification of periodontal diseases was proposed by the 2017 World Workshop, in which periodontitis is classified with stages and grades. For grading, rate of progression is evaluated by longitudinal data of radiographic bone loss or clinical attachment level, % bone loss/age, balance of biofilm deposits, and levels of destruction^51–55^. A case with lesser biofilm, but severe bone loss occurring at young age, is estimated to be at high risk for periodontal destruction with a poor response to periodontal treatment. Elucidation of the mechanism of rapid periodontal destruction is required, and our study highlights that DAMPs may provide clues to prevent rapid periodontal destruction.

In conclusion, RNA-seq analysis demonstrated innate immune response to be related to the burst-destruction of periodontal tissues in mouse model of ligature-induced periodontitis. Moreover, DAMPs, especially S100A8 and S100A9, may be important for alveolar bone resorption.

## Methods

### Animals

Wild type male C57BL/6 J mice were purchased from the Sankyo Laboratory (Tokyo, Japan). Mice were housed in ventilated cages and 9-week-old mice were used for experiments. All mouse experiments were conducted according to the Guidelines for Proper Conduct of Animal Experiments, Science Council of Japan, and the Guideline for the Care and Use of Laboratory Animals at Tokyo Medical and Dental University. All experimental protocols involving animal use and euthanasia were approved (A2017-323A and A2018-300A) by the Animal Care Committee of the Experimental Animal Center in Tokyo Medical and Dental University.

### Ligature-induced periodontitis model

Gingival inflammation and loss of alveolar bone were induced by ligation of the 2nd molar tooth in mice, as previously described^9,56^. A 6-0 silk ligature was placed around the maxillary left second molar in 9-week-old male mice under anesthesia using intraperitoneal injection of 400 mg/kg chloral hydrate^56^. The contralateral 2nd molar tooth in each mouse was kept unligated. *In-vivo* micro CT imaging (R_mCT2, Rigaku Corp.) was employed after anesthesia by isoflurane inhalation (induction with 4% and maintenance using 2%). Imaging was performed before ligation and at 1, 3, 5, and 8 days after ligation using a micro CT unit with a field of view of 5 mm diameter × 5 mm height at conditions of 90 kV tube voltage and 160 μA tube current for 3 min. The mice with displaced ligature during experimental period were excluded from the evaluation. Bone loss volume was evaluated using micro CT imaging and OsiriX Lite ver. 9.5.1 (Pixmeo, Geneva, Switzerland). The mice were sacrificed by isoflurane overdose and periodontal tissue samples were collected.

### Quantitative PCR

Gingival tissue was taken from around the maxillary second molars at 1, 3, and 7 days after ligation and homogenized. Total RNA was extracted from the excised gingival tissue using the NucleoSpin® RNA kit (TaKaRa Bio, Shiga, Japan) and quantified by measuring the absorbance at 260 nm. Isolated RNA (500 ng) was reverse transcribed using PrimeScript^TM^ RT Master Mix (TaKaRa Bio), and qPCR was performed with cDNA using the Thermal Cycler Dice® Real Time System II (TaKaRa Bio). PCR mixtures were prepared by SYBR® Premix Ex Taq™ II (TaKaRa Bio), and PCR conditions were set according to the manufacturer’s protocol (TaKaRa Bio). The sequences of sense and antisense primers used for quantification of gene expression by qPCR are listed in Supplementary Table S2. Gene expression levels were normalized to those of the reference gene *36b4* or *GAPDH*.

### RNA sequencing

Extraction of total RNA at 3 days post-ligation was performed by a combination of TRIzol and NucleoSpin® RNA kit, and 500 ng of total RNA was used as the starting material. After deletion of rRNA using TruSeq Stranded Total RNA with Ribo-Zero Gold kit (Illumina, San Diego, USA), RNA-seq libraries were prepared using ScriptSeq Complete Kit, ScriptSeq Index PCR Primers Set, and MiSeq Reagent Kit (15 Gbp) (Illumina), according to manufacturer’s instructions. The adjusted libraries were sequenced with 2 × 101-bp paired-end reads on the Illumina MiSeq sequencer. RNA-seq data, obtained in this study, are available in the DNA Data Bank of Japan (http://www.ddbj.nig.ac.jp/) under accession number DRA007913. Raw sequencing read data was checked and visualized for quality using FastQC (v0.11.4). The p reads were aligned to mm10 using Bowtie2 (v. 2.2.6)/TopHat2 (v. 2.1.0) software^57^. Uniquely mapped reads were normalized to gene expression as FPKM using Cufflinks (v. 2.2.1) software package^58^. Cufflinks was used to detect DEGs between ligated and unligated samples and Cuffdiff was used to calculate the fold change for every gene, *p*-value, and *q*-value by the FDR with Benjamini-Hochberg correction. DEGs were defined when the *q*-value was <0.05 and fold change of FPKM was ≥2.0. The mouse reference genome and RefSeq annotation were downloaded from UCSC Genome Browser (https://genome.ucsc.edu/). Enriched GO terms were analyzed using the Database for Annotation, Visualization and Integrated Discovery (DAVID, https://david.ncifcrf.gov/) with the annotation dataset for GO biological process. Information about specific GO terms was obtained from the NCBI database (ftp://ftp.ncbi.nlm.nih.gov/gene/DATA/). Most of the data were corrected and calculated by custom Perl scripts. Plots and graphs obtained from RNA-seq analysis were visualized using ggplot2, cummeRbund, and other R packages (ver. 3.5.1). Clustering analysis was conducted by the function hclust of the R software.

### Histological analysis of gingival tissue

Skin of the upper jaw at 3 and 8 days post-ligation was de-fleshed and fixed in 4% paraformaldehyde in phosphate-buffered saline for 24 h. The maxillae were decalcified by 150 mM EDTA in phosphate-buffered saline for 14 days at 4 °C and then embedded in paraffin. Serial sections (5-μm-thick) were then prepared and stained with HE. TRAP and ALP staining was performed using the TRAP and ALP Double Stain Kit (Wako Pure Chemical Industries, Ltd., Osaka, Japan). After washing the sections with distilled water, they were incubated with TRAP staining solution and ALP staining solution for 30 min, respectively. Samples were directly embedded with mounting reagent (Malinol 750 cps, Muto Pure Chemical, Tokyo, Japan) after washing with distilled water.

Immunohistochemistry was performed to detect the expression of S100A8 in the gingiva. The paraffin-embedded sections were de-paraffinized and subjected to antigen retrieval in TE buffer (pH 9.0) with 0.05% Tween 20 (Sigma-Aldrich Japan, Tokyo, Japan) in a microwave oven. The sections were pre-incubated for 30 min with 1% bovine serum albumin in PBS at room temperature. The sections were subsequently incubated with goat polyclonal anti-S100A8 antibody (1:500, R&D systems, Minneapolis, MN, USA) overnight at 4 °C, followed by incubation with secondary antibody (anti-goat IgG antibody labeled with Alexa 488 (1:500 dilution; Molecular Probes, Grand Island, NY, USA)) for 1 h at room temperature. All sections were stained with the nuclear counterstain 4′,6-diamidino-2-phenylindole dihydrochloride (DAPI, Dojindo, Kumamoto, Japan), and images were taken using the EVOS® Floid Cell Imaging station (Thermo Fisher Scientific, MA, USA). Quantitative analyses were performed using Adobe Photoshop CC 2019 software (San Jose, CA, USA).

### Cell cultures

Ca9-22 cell line was purchased from the Japanese Collection of Research Bioresources Cell Bank (Osaka, Japan). The cells were cultured in High-glucose Dulbecco’s Modified Eagle’s Medium (DMEM; Wako, Osaka, Japan) supplemented with 10% fetal bovine serum (FBS; Gibco, Tokyo, Japan) at 37 °C in a humidified atmosphere of 5% CO_2_ and 95% air. Cells in the logarithmic growth phase were used for the experiments.

### Small interfering RNA (siRNA) transfection

siRNAs targeting S100A8 (ON-TARGET plus Human S100A8 (6279) siRNA, #7, #8) and S100A9 (ON-TARGET plus Human S100A9 (6280) siRNA, #5, #8), as well as the non-targeting control siRNA (ON-TARGET plus Non-targeting Pool) were purchased from Dharmacon (Dharmacon, Horizon Discovery, UK). The sequences of siRNA for S100A8, S100A9, and non-target negative control are listed in Supplementary Table S3. The Ca9-22 cells were seeded in 12-well plates at a density of 1 × 10^5^ cells/well and cultured for 24 h in antibiotic-free DMEM containing 10% FBS. siRNA (100 nM) was mixed with 2 μL of FuGENE HD transfection reagent (Promega Corporation, Madison, WI, USA) in 500 μL of Opti-MEM Reduced-Serum Medium (Gibco). The mixture was incubated for 20 min at room temperature, and added to the cell culture. After 6 h, 500 μL of DMEM containing 10% FBS was added and cultured again for 48 h. The cells were treated with 5 ng/ml TNF-α for 24 h after 12-h starvation (DMEM containing 0.1% BSA). Total RNA was extracted with Nucleospin RNA kit, and reverse transcription PCR was performed with Primescript RT mix.

### Statistical analysis

Values are expressed as mean and standard error (SE). Paired *t-*test with Bonferroni correction was performed to evaluate differences between unligated and ligated sites. Unpaired *t-*test with Bonferroni correction was performed to compare mRNA expressions at ligated site of gingiva at different experimental time points. *In vitro* study, ANOVA followed Dunnett’s test was performed for comparison of the effect of siRNA. Statistical analyses were performed using SPSS software, version 22.0 (SPSS, Inc., Chicago, IL, USA). A value of *p* < 0.05 was considered statistically significant.

## Supplementary information

Supplementary Information
