# Supplementary material for: RNA sequencing for ligature induced periodontitis in mice revealed important role of S100A8 and S100A9 for periodontal destruction
Source: Sci Rep. 2019 Oct 11;9:14663. doi: 10.1038/s41598-019-50959-7 (PMC6789140; doi:10.1038/s41598-019-50959-7)
Supplement: Supplementary file 1 — Supplementary Information [file 41598_2019_50959_MOESM1_ESM.pdf]

Supplementary Information

RNA sequencing for ligature induced periodontitis in mice revealed important role of S100A8 and S100A9 for periodontal destruction

Shogo Maekawa, Satoru Onizuka, Sayaka Katagiri, Masahiro Hatasa, Yujin Ohsugi, Naoki Sasaki, Kazuki Watanabe, Anri Ohtsu, Rina Komazaki, Kohei Ogura, Tohru Miyoshi-Akiyama, Takanori Iwata, Hiroshi Nitta, Yuichi Izumi

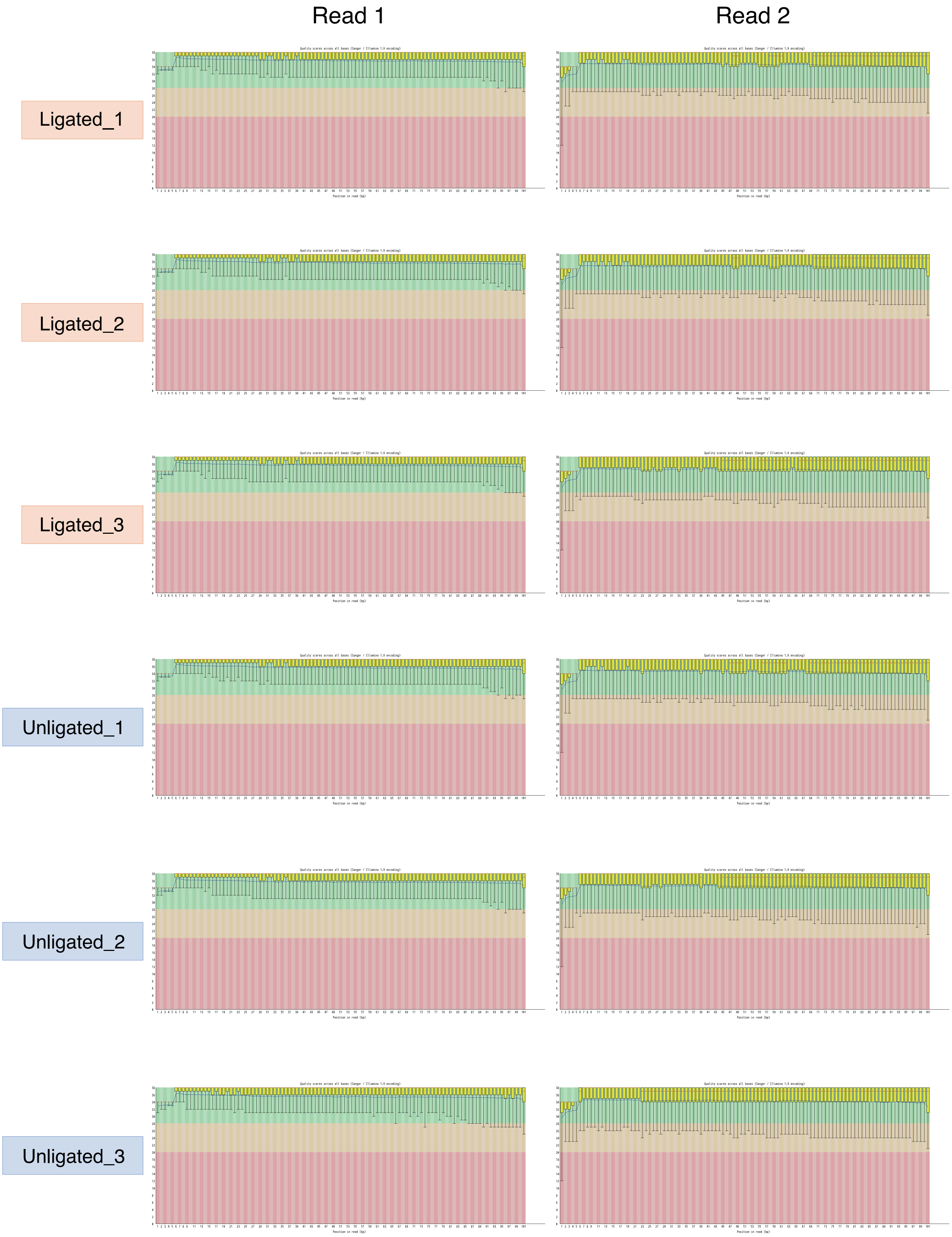

Supplementary Figure S1: Quality of sequenced reads for each base viewed using FastQC software.

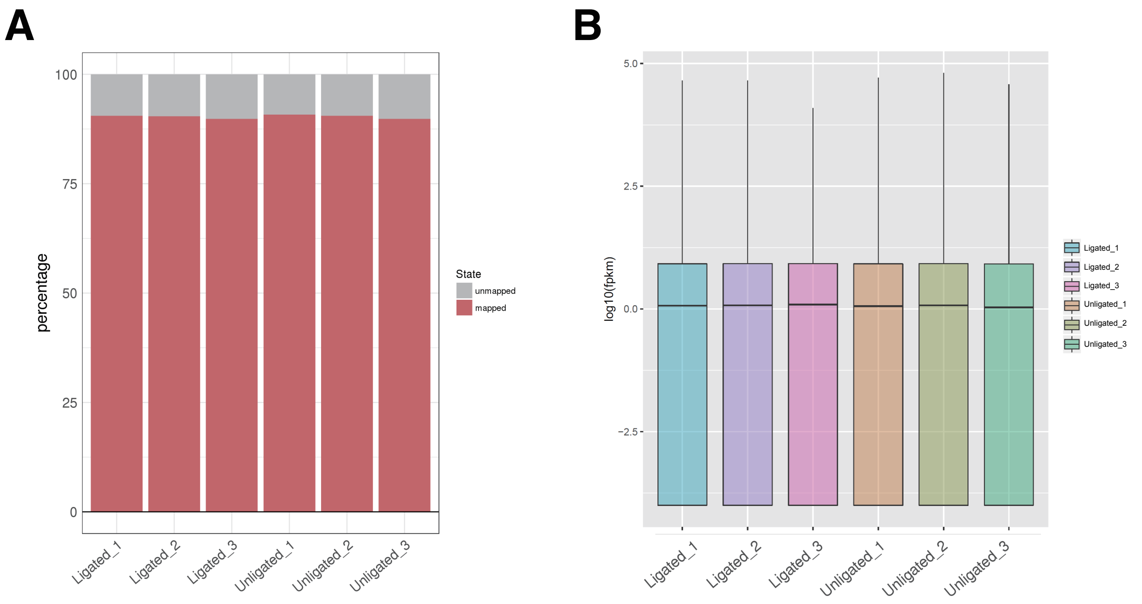

Supplementary Figure S2: Mapping rate of each sample and FPKM distribution among gingiva. Mapping rate of each sample was obtained from TopHat2. The red and gray bars represent the rate of mapped- and unmapped-reads, respectively (A). The FPKM distribution (log 10 scale) among 6 samples was analyzed by cummeRbund package (B).

The genes that showed fold change ≥ 2, *q*-value < 0.05

Up-regulated DEGs in ligated gingiva

Entrez_ID	gene_name	description
17082	*Il1rl1*	interleukin 1 receptor-like 1
12835	*Col6a3*	collagen, type VI, alpha 3
14598	*Ggt1*	gamma-glutamyltransferase 1
58223	*Mmp19*	matrix metallopeptidase 19
12822	*Col18a1*	collagen, type XVIII, alpha 1
22271	*Upp1*	uridine phosphorylase 1
14230	*Fkbp10*	FK506 binding protein 10
17896	*Myl4*	myosin, light polypeptide 4
80879	*Slc16a3*	solute carrier family 16 (monocarboxylic acid transporters), member
20308	*Ccl9*	chemokine (C-C motif) ligand 9
20716	*Serpina3n*	serine (or cysteine) peptidase inhibitor, clade A, member 3N
13371	*Dio2*	deiodinase, iodothyronine, type II
320581	*Idi2*	isopentenyl-diphosphate delta isomerase 2
20379	*Sfrp4*	secreted frizzled-related protein 4
80838	*Hist1h1a*	histone cluster 1, H1a
12257	*Tspo*	translocator protein
14962	*Cfb*	complement factor B
16948	*Lox*	lysyl oxidase
15957	*Ifit1*	interferon-induced protein with tetratricopeptide repeats 1
27060	*Tcirg1*	T cell, immune regulator 1, ATPase, H+ transporting, lysosomal V0
353156	*Egfl7*	EGF-like domain 7
23796	*Aplnr*	apelin receptor
17395	*Mmp9*	matrix metallopeptidase 9
16176	*Il1b*	interleukin 1 beta
20201	*S100a8*	S100 calcium binding protein A8 (calgranulin A)
13038	*Ctsk*	cathepsin K
13040	*Ctss*	cathepsin S
11853	*Rhoc*	ras homolog family member C
20202	*S100a9*	S100 calcium binding protein A9 (calgranulin B)
16792	*Laptm5*	lysosomal-associated protein transmembrane 5
12260	*C1qb*	complement component 1, q subcomponent, beta polypeptide
269642	*Nat8l*	N-acetyltransferase 8-like
20311	*Cxcl5*	chemokine (C-X-C motif) ligand 5
57349	*Ppbp*	pro-platelet basic protein
330122	*Cxcl3*	chemokine (C-X-C motif) ligand 3
14825	*Cxcl1*	chemokine (C-X-C motif) ligand 1
381651	*Gm1045*	predicted gene 1045
15891	*Ibsp*	integrin binding sialoprotein
20750	*Spp1*	secreted phosphoprotein 1
109900	*Asl*	argininosuccinate lyase
17969	*Ncf1*	neutrophil cytosolic factor 1
56847	*Aldh1a3*	aldehyde dehydrogenase family 1, subfamily A3
12721	*Coro1a*	coronin, actin binding protein 1A
12827	*Col4a2*	collagen, type IV, alpha 2
18791	*Plat*	plasminogen activator, tissue
12826	*Col4a1*	collagen, type IV, alpha 1
17386	*Mmp13*	matrix metallopeptidase 13
17392	*Mmp3*	matrix metallopeptidase 3
15894	*Icam1*	intercellular adhesion molecule 1
12772	*Ccr2*	chemokine (C-C motif) receptor 2
102644	*Oaf*	out at first homolog
12816	*Col12a1*	collagen, type XII, alpha 1
21857	*Timp1*	tissue inhibitor of metalloproteinase 1
16186	*Il2rg*	interleukin 2 receptor, gamma chain

Down-regulated DEGs in ligated gingiva

Entrez_ID	gene_name	description
110257	*Hba-a2*	hemoglobin alpha, adult chain 2
110257	*Hba-a2*	hemoglobin alpha, adult chain 2
17882	*Myh2*	myosin, heavy polypeptide 2, skeletal muscle, adult
53313	*Atp2a3*	ATPase, Ca++ transporting, ubiquitous
23795	*Agr2*	anterior gradient 2
238564	*Mylk4*	myosin light chain kinase family, member 4
18095	*Nkx3-1*	NK-3 transcription factor, locus 1 (Drosophila)
74843	*Mss51*	MSS51 mitochondrial translational activator
331063	*Gsdmc2*	gasdermin C2
57277	*Slurp1*	secreted Ly6/Plaur domain containing 1
19293	*Pvalb*	parvalbumin
13184	*Dcpp1*	demilune cell and parotid protein 1
630537	*Dcpp2*	demilune cell and parotid protein 2
620253	*Dcpp3*	demilune cell and parotid protein 3
21785	*Tff2*	trefoil factor 2 (spasmolytic protein 1)
320092	*E030003E1*	RIKEN cDNA E030003E18 gene
66557	*Bpifb2*	BPI fold containing family B, member 2
12096	*Bglap*	bone gamma carboxyglutamate protein
74180	*Muc5b*	mucin 5, subtype B, tracheobronchial
67133	*Gp2*	glycoprotein 2 (zymogen granule membrane)
12862	*Cox6a2*	cytochrome c oxidase subunit VIa polypeptide 2
11656	*Alas2*	aminolevulinic acid synthase 2, erythroid

Supplementary Table S1: The table shows 78 (55 up-regulated and 23 down-regulated) DEGs

	gene	primers	
		Sense (5'-3')	Antisense(5'-3')
Mus musculus	***36b4***	GCTCCAAGCAGATGCAGCA	CCGGATGTGAGGCAGCAG
	S100a8	AAATCACCATGCCCTCTACAAG	CCCACTTTTATCACCATCGCAA
	S100a9	ATACTCTAGGAAGGAAGGACACC	TCCATGATGTCATTTATGAGGGC
	Il1b	GCAACTGTTCCTGAACTCAACT	ATCTTTTGGGGTCCGTCAACT
	Ctsk	ACAGTAGCCACGCTTCCTATC	ACTGCTTCTGGTGAGTCTTCTT
	Mmp9	CTGGACAGCCAGACACTAAAG	CTCGCGGCAAGTCTTCAGAG
	Mmp3	ACATGGAGACTTTGTCCCTTTTG	TTGGCTGAGTGGTAGAGTCCC
	Mmp19	CTGTGGCTGGCATTCTTACTT	GGGCAGTCCAGATGCTTCC
	Timp1	GCAACTCGGACCTGGTCATAA	CGGCCCGTGATGAGAAACT
	Ccl9	CCCTCTCCTTCCTCATTCTTACA	AGTCTTGAAAGCCCATGTGAAA
	Ncf1	TTCTTCAAAGTGCGGCCTGAT	CAGCTACGTTATTCTTGCCATCT
	Spp1	AGCAAGAAACTCTTCCAAGCAA	GTGAGATTCGTCAGATTCATCCG
		Sense (5'-3')	Antisense(5'-3')
Homo sapiens	***GAPDH***	GGAGCGAGATCCCTCCAAAAT	CTCCTTAATGTCACGCACGAT
	S100A8	GGGATGACCTGAAGAAATTGCTA	TGTTGATATCCAACTCTTTGAACCA
	S100A9	GTGCGAAAAGATCTGCAAAATTT	GGTCCTCCATGATGTGTTCTATGA
	IL1B	ATGATGGCTTATTACAGTGGCAA	GTCGGAGATTCGTAGCTGGA
	CTSK	AGCGATAATCTGAACCATGCAGT	ATGCCACAGGCGTTGTTCTTA

Supplementary Table S2: Primers used for quantitative PCR analysis in this study.

siRNA	Dharmacon Ref#: S0-2776848G
	Target Sequence
D-0018-10-10-05 ON-TARGETplus Non-targeting Pool	UGGUUUACAUGUCGACUAA, UGGUUUACAUGUUGUGUGA, UGGUUUACAUGUUUUCUGA, UGGUUUACAUGUUUUCCUA
LQ-011770-00-0002, ON-TARGETplus Human S100A8 (6279) siRNA	
siRNA J-011770-07, S100A8	GGGAUGACCUGAAGAAAUU
siRNA J-011770-08, S100A8	GCAGUUAACUUCCAGGAGU
LQ-011384-00-0002, ON-TARGETplus Human S100A9 (6280) siRNA	
siRNA J-011384-05, S100A9	GGUCAUAGAACACAUCAUG
siRNA J-011384-08, S100A9	ACACAAAUGCAGACAAGCA

Supplementary Table S3: Sequence of siRNA in this study.
